# The prevalence of metabolic syndrome and cardiovascular risk factors in adults in southern China

**DOI:** 10.1186/1471-2458-12-64

**Published:** 2012-01-21

**Authors:** Xiang Qian Lao, Yong Hui Zhang, Martin Chi Sang Wong, Yan Jun Xu, Hao Feng Xu, Shao Ping Nie, Wen Jun Ma, G Neil Thomas, Ignatius Tak Sun Yu

**Affiliations:** 1 School of Public Health and Primary Care, Faculty of Medicine, The Chinese University of Hong Kong, HongKong, China; 2 Center for disease Control and Prevention of Guangdong Province, Guangzhou, China; 3 Guangdong Institute of Public Health, 176, Xin Gang Xi Road, Guangzhou, China; 4 School of Health and Population Sciences, The University of Birmingham Edgbaston, Birmingham, UK

## Abstract

**Background:**

The metabolic syndrome has been shown to increase the incidence of cardiovascular disease. Little information exists on the prevalence of the metabolic syndrome for southern Chinese. We therefore investigate the prevalence of the metabolic syndrome in a southern Chinese population with 85 million residents.

**Methods:**

The Guangdong Nutrition and Health Survey 2002 is a cross-sectional survey designed to assess the health and nutritional status of 85 million residents in Guangdong province located in southern China. Stratified multistage random sampling method was applied in this survey and a provincial representative sample of 6,468 residents aged 20 years or above was obtained in the present study. The participants received a full medical check-up including measurement of blood pressure, obesity indices, fasting lipids and glucose levels. Data describing socioeconomic and lifestyle factors was also collected through interview. Metabolic syndrome was defined in accordance with the International Diabetes Federation criteria.

**Results:**

The prevalence of metabolic syndrome was 7.30%, translating into a total of 4.0 million residents aged 20 years or above having the condition in this southern Chinese population. The urban population had higher prevalence of the syndrome than the rural population (10.57% vs 4.30%). Females had a higher prevalence of metabolic syndrome than males (8.99% vs 5.27%). More than 60% of the adults had at least one component of the metabolic syndrome.

**Conclusions:**

Our results indicate that a large proportion of southern Chinese adults have the metabolic syndrome and associated risk factors. The metabolic syndrome has become an important public health problem in China. These findings emphasize the urgent need to develop population level strategies for the prevention, detection, and treatment of cardiovascular risk in China.

## Background

Cardiovascular disease is the leading cause of mortality in China as well as worldwide [1,2]. The constellation of cardiovascular risk factors including abdominal obesity, raised fasting glucose level and dislipidemia is termed the metabolic syndrome (MetSyn). The MetSyn has been associated with increased risk of diabetes, cardiovascular disease and subsequent mortality. In recent decades with its rapid economic growth and aging population China has experienced a cardiovascular disease epidemic.

As China is a country with a population of 1.3 billion living in a large geographical area, the variations in demographic characteristics, cultural behaviors and lifestyle habits vary substantially in different regions. This may result in differing disease prevalences in different populations. A nationwide survey by Gu et al has shown that the prevalence of MetSyn varied geographically in China [3]. The prevalence of obesity component in northern China was shown to be two-fold higher than that in southern China [3]. Data on the MetSyn prevalence for different populations with relatively homogeneous characteristics are necessary for developing prevention strategies at population level. There were quite a number of studies concerning the prevalence of the MetSyn among Chinese in the last decade [3-7]. However, most of these studies are small-scale, investigations. They either focused on special groups, such as professionals or elderly, or were restricted in one or several small communities. Large scale, representative, population-based survey in China is rare, especially for southern Chinese.

Guangdong is the province located in southern China with a population of 85 million permanent residents. Its capital is Canton, which is about 180 kilometers north of Hong Kong. Cantonese people have relatively unique culture and lifestyle as compared to northern Chinese. In addition, Guangdong is the first province where the Chinese leader Deng Xiao Ping started the economic reform and open policy in 1979. When compared with other inland provinces, Guangdong is more economically developed and urbanized. In order to elucidate the present situation of the MetSyn in this Cantonese population, we report its prevalence based on the data from the Guangdong Nutrition and Health Survey 2002 (GNHS 2002) [8].

## Methods

The Guangdong Nutrition and Health Survey (GNHS) 2002 was conducted by the Guangdong Province Center for Disease Control and Prevention (CDC) and the Health Bureau of Guangdong Province in 2002. The GNHS 2002 corresponded with the China National Nutrition and Health Survey (CNNHS) 2002 [9-11], which covered 31 provinces, autonomous regions, and municipalities nationwide. Based on the CNNHS 2002 sampling protocols, eight urban areas or rural villages in Guangdong province were required to be sampled as a part of the CNNHS 2002. In order to have sufficient statistical power to analyze the provincial data independently and to obtain more reliable health information for the provincial population as a whole, five additional urban areas or rural villages were sampled in the GNHS 2002. Therefore, the GNHS 2002 has a representative provincial population sample from a total of 13 urban areas or rural villages (six urban city districts and seven rural villages) [8]. Ethics approval was obtained from the Ethics Committee of China Centre for Disease Control. All participants gave informed consent prior to the survey.

The sampling methods and survey protocols as well as the quality control for the GNHS 2002 were similar to those for the CNNHS 2002 [9-11]. The stratified multistage cluster sampling with probability proportional to size method was used. Briefly, the cities and counties of the province were classified into four strata (large cities; small to medium cities; class 1 and class 2 rural areas) based on their economic development levels identified by the central government of China. The first stage systemic sampling was conducted in each stratum: three districts from the large cities, three districts from the small to medium cities, four counties from the class 1 rural areas and three counties from the class 2 rural counties were randomly selected based on the population size. The second stage sampling was subsequently conducted in each selected districts or counties: three neighborhoods (urban) or townships (rural) were sampled from each selected districts or counties using the same systematic random sampling methods as that in the first stage. In the third stage, two residential committees (urban) or villages (rural) were sampled from each selected neighborhood or townships using the same systematic random sampling methods as those in the first and second stages. In the fourth stage, around 90 households with a record in the household registration system from each residential committees or villages were randomly sampled (the households in the registration system generally do not include migrant workers). All members of the selected household were invited to participate in the health survey, including a questionnaire interview and a general health examination. The overall response rate for the health survey was 89.45%. A total of 78 residential committees or villages and 7,180 households were finally included in the GNHS 2002 with 25,459 participants, representing the Guangdong provincial permanent residential population of 85,221,747 in 2000 [12]. In addition to the general questionnaire interview and health examination, a third of the sampled households (2,424 households with 9,509 all age residents) were randomly selected for dietary intake examination, as well as blood sample laboratory test including fasting glucose and lipids profiles. Therefore, a total of 6,468 participants aged 20 years or above were included in the present analysis.

A central survey site was set up in each residential committee or village and the participants were required to be interviewed and receive the health examination on-site. All interviews and examinations following standardized protocols were conducted by physicians who received training specifically for the GNHS 2002. The questionnaire interview collected a wide range of information including demographic characteristics, life style and family and personal disease histories. Weight and height were measured with light indoor clothing and without shoes. Waist circumference measurement was made at minimal inspiration to the nearest 0.1 cm, midway between the lowest rib and the superior border of the iliac crest. Both weight and waist were measured in the morning before breakfast.

The blood pressure measurement was based on the 1999 World Health Organization/International Society of Hypertension guidelines on hypertension [13]. Two consecutive readings of the blood pressure on right arms were taken after the participant in a seated position for 5 minutes rest. The average of the two readings was used for analysis.

Blood samples were drawn in the morning after an overnight fast using vacutainer tubes. Plasma glucose was measured within three hours after obtaining the blood sample using a spectrophotometer 721/722. For those participants with a plasma glucose level of 5.50 mmol/l or above, an Oral Glucose Tolerance Test (OGTT) was performed on the subsequent sixth day. Additional plasma samples were stored in airtight tubes at -80°C prior to shipment on dry ice to the CDC for the measurement of the lipids. Total cholesterol, triglyceride and HDL-cholesterol were determined using a Hitachi 7060 Automatic Chemical Analyzer in the CDC laboratory.

The International Diabetes Federation criteria (IDF) were used to define MetSyn in the present study because this definition considers the ethnic difference for central obesity. According to the IDF criteria, participants are classified as having MetSyn if they have central obesity (waist circumference > 90 cm for men and > 80 cm for women) plus any other two abnormalities of those shown below:

1. Hypertension: systolic blood pressure ≧ 130 mmHg, Or diastolic blood pressure ≧ 85 mmHg, or treatment of previously diagnosed hypertension;

2. Hypertriglyceridemia: ≧ 1.7 mmol/l or specific medical treatment for this lipid abnormality;

3. Hypo-HDL-cholesterol: < 1.03 mmol/l for males or < 1.29 mmol/l for females;

4. Raised fasting glucose: overnight ≧5.6 mmol/l.

The present study included all men and women aged 20 years or older. All data analyses were performed using SAS software, version 9.2 (SAS Institute, Cary, NC, USA). In a manner, similar to our previous survey analysis for the U.S. National Health and Nutrition Examination Survey (NHANES) [14], the GNHS 2002 adopted a stratified multistage cluster sampling design. As such, the survey design parameters including weight, stratum and cluster were incorporated into all analyses. The weight was derived based on the provincial 2000 census data and associated administrative data. These weights account for the stratified multistage and the unequal selection probability survey design. The non-response information was also incorporated into the weight. PROC SURVEYMEANS and PROC SURVEYFREQ were used for the calculation of means and prevalence. All means and prevalence calculated in this study represented the overall estimates for the corresponding population aged 20 years or above in Guangdong province. PROC SURVEYREG and PROC SURVEYLOGISTIC were used to assess the differences between categories. Two sided *p *values of less than 0.05 were considered statistically significant. 95% confidence intervals were calculated and presented in the present study. Domain statement was used for the subpopulation analyses.

## Results

The GNHS 2002 included a representative sample of 6,468 residents aged 20 years or above with biochemical test results. The mean age of the population was 44.91 (43.63, 46.19). Table 1 presents the risk factors for MetSyn among the population stratified by sex and region. Compared to rural population, participants in the urban population were older and had higher BMI, waist circumference, total cholesterol, triglyceride and fasting glucose levels. The female population in the urban settings was more educated than those in the rural settings, while male participants in rural regions consumed more tobacco than in urban areas. There were no significant differences between urban and rural populations for blood pressure and HDL-cholesterol, or for alcohol consumption. Compared to females, male participants generally had a higher waist circumference, diastolic blood pressure, triglycerides, as well as a lower HDL-cholesterol level. Male participants were more educated and consumed much more tobacco and alcohol than the females.

**Table 1:** Anthropometric, blood pressure and plasma biochemical characteristics in the population aged 20 years or above by sex and region

		Male (3,148)	Female (3,320)	*P *
Age (years)	Urban	47.70 (45.24, 50.16)	46.35 (44.14, 48.56)	< 0.001

	Rural	43.47 (41.83, 45.11)	43.18 (41.64, 45.11)	0.68

	*p*	0.010	0.026	

Body Mass Index (kg/m^2^)	Urban	23.29 (22.83, 23.75)	23.03 (22.83, 23.23)	0.28

	Rural	21.05 (20.29, 21.82)	21.02 (20.63, 21.41)	0.89

	*p*	< 0.001	< 0.001	

Waist circumference (cm)	Urban	80.61 (79.80, 81.45)	74.52 (72.19, 76.86)	< 0.001

	Rural	74.95 (73.62, 76.27)	71.71 (71.03, 72.40)	< 0.001

	*p*	< 0.001	0.028	

Systolic blood pressure (mmHg)	Urban	123.48 (120.79, 126.16)	119.93 (116.65, 123.20)	0.002

	Rural	119.52 (115.57, 123.47)	117.57 (111.52, 123.62)	0.25

	*p*	0.094	0.46	

Diastolic blood pressure (mmHg)	Urban	78.43 (77.04, 79.81)	74.34 (72.83, 75.85)	< 0.001

	Rural	76.76 (75.77, 77.74)	74.52 (73.39, 75.65)	0.002

	*p*	0.053	0.84	

Total cholesterol (mmol/L)	Urban	4.52 (4.41, 4.64)	4.55 (4.41, 4.70)	0.43

	Rural	4.19 (4.01, 4.37)	4.14 (4.03, 4.25)	0.32

	*p*	0.007	< 0.001	

HDL-cholesterol (mmol/L)	Urban	1.27 (1.15, 1.34)	1.38 (1.31, 1.44)	< 0.001

	Rural	1.32 (1.26, 1.38)	1.40 (1.32, 1.48)	0.028

	*p*	0.20	0.63	

Triglyceride (mmol/L)	Urban	1.37 (1.25, 1.49)	1.17 (1.02, 1.32)	0.001

	Rural	1.05 (0.95, 1.15)	0.96 (0.93, 1.00)	0.034

	*p*	0.001	0.015	

Fasting glucose (mmol/L)	Urban	5.08 (4.95, 5.21)	5.10 (4.97, 5.23)	0.25

	Rural	4.90 (4.75, 5.06)	4.81 (4.67, 4.94)	0.019

	*p*	0.078	0.006	

Education (no formal school education)	Urban	97.90 (96.34, 99.46)	90.21 (85.47, 94.94)	< .0001

	Rural	97.48 (96.42, 98.53)	83.49 (78.41, 88.57)	< .0001

	*p*	0.63	0.037	

Tobacco consumptions (ever)	Urban	45.53 (39.34, 51.72)	2.33 (1.08, 3.58)	< 0.001

	Rural	57.49 (50.00, 64.98)	0.86 (0.00, 2.11)	< 0.001

	*p*	0.006	0.14	

Alcohol consumptions (ever)	Urban	33.84(31.15, 36.53)	4.40 (2.28, 6.53)	< 0.001

	Rural	30.56 (25.38, 35.75)	4.45 (3.04, 5.86)	< 0.001

	*p*	0.21	0.97	

Values were presented in mean (95% CI) for continuous variables and prevalence (95% CI) for categorical variables

Smoking in the present study was defined as currently smoking or having at least one cigarette daily for six months or above.

Alcohol consumption was defined as currently or once having one drink at least once per week over the last 12 months.

The prevalence of the MetSyn in this population was 7.30%. Table 2 presents the prevalence of the MetSyn as well as the individual components among the population stratified by regions and sex. Compared to the rural population, the urban population had higher prevalence of the MetSyn. The urban population generally had higher prevalence of most individual components, except for low HDL-cholesterol levels. Compared to females, male residents had lower prevalence of central obesity and low HDL-cholesterol, but a higher prevalence of hypertension and high triglycerides. There was no significant sex difference in the prevalence of raised fasting glucose.

**Table 2:** Prevalence of individual components of the metabolic syndrome based on International Diabetes Federation guidelines in southern Chinese aged 20 years or above

		Male	Female	*p*
Central obesity	Urban	16.58 (13.51, 19.65)	25.50 (15.94, 35.07)	0.003

	Rural	6.56 (3.90, 9.22)	15.35 (12.66, 18.05)	< 0.001

	*p*	< 0.001	0.008	

Hypertension	Urban	40.45 (32.81, 48.09)	31.52 (25.20, 37.83)	< 0.001

	Rural	27.26 (19.82, 34.70)	24.62 (15.19, 34.05)	0.16

	*p*	0.006	0.19	

hypertriglyceridaemia	Urban	24.52 (18.16, 30.88)	17.37 (9.46, 25.29)	0.006

	Rural	8.92 (7.06, 10.77)	5.61 (4.90, 6.33)	< 0.001

	*p*	< 0.001	< 0.001	

Low HDL-cholesterol	Urban	25.22 (12.88, 37.57)	40.89 (35.27, 46.51)	0.002

	Rural	16.04 (11.03, 21.05)	40.40 (28.96, 51.85)	< 0.001

	*p*	0.088	0.93	

Hyperglycaemia:	Urban	14.36 (10.95, 17.78)	13.55 (10.75, 16.35)	0.56

	Rural	9.52 (2.37, 16.65)	7.26 (2.93, 11.59)	0.053

	*p*	0.2273	0.0222	

MetSyn	Urban	8.56 (7.82, 9.30)	12.18 (9.25, 15.11)	< 0.001

	Rural	2.45 (1.12, 3.79)	5.94 (2.95, 8.92)	0.005

	*p*	< 0.001	0.003	

Total MetSyn in the whole population		5.27 (4.19, 6.39)	8.99 (6.84, 11.14)	0.036

**Figure 1:**
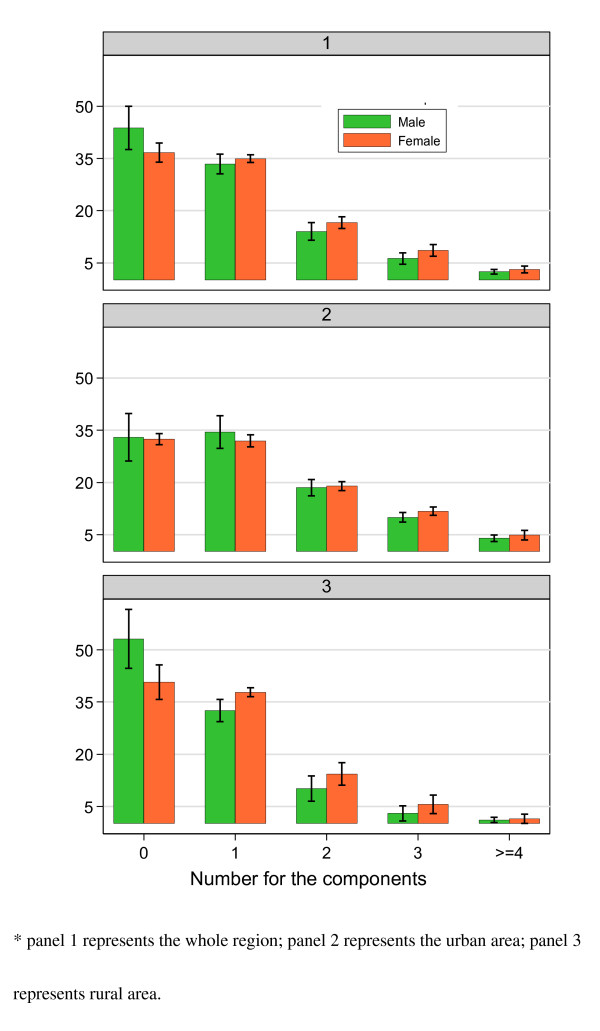
**Gender and region-specific prevalence of the International Diabetes Federation (IDF)-based metabolic syndrome components in southern China**.

Table 3 presents the prevalence of the MetSyn in the population stratified by age, sex and region. The prevalence of MetSyn generally increased significantly with increasing age. Figure 1 shows the sex and region-specific prevalence of population with one or more components of the MetSyn. A total of 60.16% of the population aged 20 years or above had at least one component of MetSyn. The proportions of having at least one component of MetSyn among urban male, urban female, rural male and rural female populations were 77.00%, 77.54%, 46.86% and 59.38%, respectively.

**Table 3:** Prevalence of the Metabolic syndrome in the population by age, sex and region

		Total (n = 6468)	Age group (years)			P for trend
				
			20-39 (n = 2942)	40-59 (n = 2277)	60-(n = 1249)	
All regions						

	Men	5.27 (4.19, 6.39)	2.70 (1.53, 3.87)	6.14 (3.80, 8.47)	7.83 (5.60, 10.05)	0.001

	Women	8.99 (6.84, 11.14)	2.18 (1.07, 3.29)	9.88 (7.38, 12.38)	21.85 (14.72, 28.99)	< 0.001

	Total	7.30 (5.78, 8.82)	2.40 (1.58, 3.22)	8.16 (6.41, 9.90)	14.69 (10.87, 18.51)	< 0.001

Urban						

	Men	8.56 (7.82, 9.30)	4.11 (2.29, 5.93)	10.39 (7.82, 12.96)	11.87 (9.33, 14.42)	< 0.001

	Women	12.18 (9.25, 15.11)	2.85 (0.99, 4.71)	12.68 (8.29, 17.07)	27.58 (20.87, 34.29)	< 0.001

	Sub total	10.57 (9.02, 12.13)	3.39 (2.03, 4.75)	11.72 (9.01,14.44)	19.60 (15.55, 23.65)	< 0.001

Rural						

	Men	2.45 (1.12, 3.79)	1.52 (0.059, 2.99)	3.09 (0.20, 5.97)	2.82 (0.01, 6.67)	0.65

	Women	5.94 (2.95, 8.92)	1.62 (0.30, 2.94)	7.15 (4.10, 10.20)	14.59 (1.01, 28.17)	< 0.001

	Sub total	4.30 (2.11, 6.49)	1.58 (0.57, 2.59)	5.14 (2.85, 7.44)	8.54 (1.53, 15.55)	0.011

## Discussion

The results of the representative, population-based GHNS 2002 show that the prevalence of the MetSyn based on the IDF criteria was common in this southern Chinese population at 7.30%. This translates into a total of 4.0 million adults having MetSyn in Guangdong province which has a population of 85 millions (around 55 million residents 20 years of age or above), with more than 60% having at least one individual component of MetSyn. The urban population had higher prevalence of MetSyn than the rural population (10.57% vs 4.30%). Female had higher prevalence of MetSyn than male (8.99% vs 5.27%).

Comparisons of the prevalence rates of MetSyn with other populations are generally difficult due to its varying definitions specified by different international authorities. The National Cholesterol Education Programme Adult Treatment Panel (NCEP ATP) III is the most widely utilized to date, especially for those studies conducted in western populations. However, it is not appropriate to use NCEP ATP III for defining central obesity for Chinese owing to the disproportionate contribution of obesity to the development of cardiovascular diseases in Asians [15-18], and thus lower thresholds of ≧ 80 or ≧ 90 cm in females and males, respectively for defining central obesity have been recommended by the WHO [19,20]. In addition to central obesity, the NCEP ATP III criterion (2001) for Hyperglycaemia is ≧ 6.1 mmol/L, while the IDF criterion is 5.6 mmol/L. Otherwise the definitions for the other three components, namely hypertriglyceridemia, hypo-HDL-cholesterol and hypertension, are the same. Nonetheless, the prevalence of MetSyn using the IDF criteria in our population (7.30%) is much lower than those of MetSyn among the American adults aged 20 years or above in NHANES III (27.9%) and NHANES 1999-2006 (34.1%) using the definition by NCEP ATP III [21]. In a study by Ford et al., they analyzed the NHANES 1999-2002 data using the IDF definition, and found the prevalence of the MetSyn among American adults was 34.5% [22].

Previous studies showed that MetSyn was prevalent in China. In the InterASIA study [3], Gu et al reported the age-standardized prevalence of MetSyn as 9·8% for men and 17·8% for women using the modified NCEP ATP III definition (the waist circumference criterion for central obesity was modified as ≧80 for female and ≧90 cm for males) in a nationwide, representative sample. They also found that the prevalence was higher in urban area than in rural area, and in female than in male, which is compatible with our findings. Yang et al compared the prevalence using the NCEP ATP III and IDF definitions in the InterASIA study, and they found that the prevalence by IDF was higher than that by NCEP ATP III (23.3% vs 16.5%) [23]. Reports on Cantonese population are relatively limited. Guangdong province was not included in the InterASIA study [3]. A study by Li et al reported the prevalence was 26.7% using the modified NCEP ATP III definition among the 1,206 participants with a mean age of 59.10 years who attended a medical examination centre [4]. The advantage of our study is that we included a representative sample targeting to the whole population with 85 million residents in Guangdong province. However, large scale survey has limitations (e.g., nonsample errors including nonresponse, coverage errors and measurement errors), which may influence the accuracy of estimation.

We previously conducted a study on MetSyn in Canton city using the IDF definition [16,24]. The prevalence of the MetSyn in that study was 25.81% in the urban participants aged 50 years above, which was higher than the prevalence of the present study (the prevalence of the MetSyn were 11.71% and 19.60% among the age group 40- and age group 60- urban populations, respectively). However, the participants of the previous study were members of a community social and welfare association, who generally have a higher social status than participants from the general population. In addition, there were more women than men in our previous study [16]. The incidence of the MetSyn increases substantially during perimenopause and early menopause [25]. Our present study showed that the prevalence of the MetSyn in women jumped from 9.88% in 40- age group to 21.85% in the 60- age group (Table 3).

The MetSyn is a constellation of cardiovascular risk factors. As the prevalence of cardiovascular risk factors continues to increase, in particular obesity, MetSyn is becoming increasingly recognized as an important public health concern worldwide [26]. In the United States, one-third of adults have MetSyn. The goal of developing the MetSyn concept is to identify individuals at high risk of cardiovascular disease or type 2 diabetes to enable targeted interventions to be introduced to prevent the development of the conditions.

## Conclusions

Our results showed that the prevalence of the MetSyn was high in this southern Chinese population. We estimate a total of 4.0 million southern Chinese adult residents have the MetSyn. However, more surveys are needed to assess the changes in trends of the prevalence of MetSyn in this population. Mozumdar et al have reported that the prevalence of the MetSyn increased from 27.9% to 34.1% during a period of around a decade in the United States [21]. China has the fastest economic development in the world. As China increases its rate of modernization and becomes more urbanized, more people are likely to have increasingly sedentary lifestyles and consumption of energy-dense diets. The prevalence of MetSyn and related cardiovascular disease is therefore expected to increase enormously. Clearly, without prevention and aggressive treatment of these conditions, the potential socioeconomic, medical and societal ramifications in China could be overwhelming. Urgent public health actions are needed to control this observed worsening situation in China.

## Competing interests

The authors declare that they have no competing interests.

## Authors' contributions

WJM and YHZ designed and oversaw the survey. YJX, HFX and SPN assisted in the planning and co-ordination of the survey. XQL designed this analysis and drafted the manuscript. WJM, CSW, GNT and ITY contributed to the interpretation of this analysis. All authors critically reviewed the manuscript for intellectual content. All authors read and approved the final manuscript.

## Acknowledgements

We thank all participants for their participation and all the staff of the survey team for their efforts that made this study possible. This survey was supported by Guangdong Science and Technology Fund (2002 C32709), Guangzhou, China; the China Ministry of Health Special Fund (2001DEA30035), Beijing, China; and the China Ministry of Science and Technology Key Research Fund (2003DIA6N008), Beijing, China.

## Pre-publication history

The pre-publication history for this paper can be accessed here:

http://www.biomedcentral.com/1471-2458/12/64/prepub
